# Experimental Phage Therapy for *Burkholderia pseudomallei* Infection

**DOI:** 10.1371/journal.pone.0158213

**Published:** 2016-07-07

**Authors:** Ong Guang-Han, Choh Leang-Chung, Kumutha Malar Vellasamy, Vanitha Mariappan, Chang Li-Yen, Jamuna Vadivelu

**Affiliations:** 1 Department of Medical Microbiology, Faculty of Medicine, University of Malaya, Kuala Lumpur, Malaysia; 2 Tropical Infectious Diseases Research and Education Centre (TIDREC), University of Malaya, Kuala Lumpur, Malaysia; University of Toledo College of Medicine and Life Sciences, UNITED STATES

## Abstract

*Burkholderia pseudomallei* is an intracellular Gram-negative bacterial pathogen intrinsically resistant to a variety of antibiotics. Phages have been developed for use as an alternative treatment therapy, particularly for bacterial infections that do not respond to conventional antibiotics. In this study, we investigated the use of phages to treat cells infected with *B*. *pseudomallei*. Phage C34 isolated from seawater was purified and characterised on the basis of its host range and morphology using transmission electron microscopy (TEM). Phage C34 was able to lyse 39.5% of *B*. *pseudomallei* clinical strains. Due to the presence of contractile tail, phage C34 is classified as a member of the family Myoviridae, a tailed double-stranded DNA virus. When 2 × 10^5^ A549 cells were exposed to 2 × 10^7^ PFU of phage C34, 24 hours prior to infection with 2 × 10^6^ CFU of *B*. *pseudomallei*, it was found that the survivability of the cells increased to 41.6 ± 6.8% as compared to 22.8 ± 6.0% in untreated control. Additionally, application of phage successfully rescued 33.3% of mice infected with *B*. *pseudomallei* and significantly reduced the bacterial load in the spleen of the phage-treated mice. These findings indicate that phage can be a potential antimicrobial agent for *B*. *pseudomallei* infections.

## Introduction

Melioidosis is a respiratory disease caused by *Burkholderia pseudomallei*, a Gram-negative bacillus bacterium. The bacteria infect both humans and animals through inhalation, ingestion and inoculation. Most of the reported cases of melioidosis were from Australia, Thailand, Singapore, Vietnam and Malaysia [1]. Meliodosis has a high mortality rate, ranging from 30–60% depending on whether the patients are septicemic [2]. The National Institute of Allergy and Infectious Disease of United States (NIAID) lists *B*. *pseudomallei* as a Category B bioterrorism agent due to the severity of infection, aerosol infectivity and worldwide availability of the bacteria.

At present, melioidosis patients are treated with a combination of antibiotics for a period of 20 weeks or longer, but the mortality is still high [3–5]. Failure to adhere to the complete 20-weeks of therapy may raise the risk of relapse [6, 7]. *B*. *pseudomallei* is resistant to many first and second generation antibiotics including cephalosporins, penicillins, macrolides, colistin, rifamycins, and aminoglycosides [8–10]. Currently, there are also no vaccines available for melioidosis [11]. The intracellular lifestyle of the bacterium following its entry into the host system leads to either an acute or chronic infection, which encompasses latency and recrudescence. This complicates the development of an efficient vaccine. Due to these complications, development of a novel antimicrobial agent against *B*. *pseudomallei* infections is vital, and the use of phages could be an alternative treatment therapy.

Bacteriophages or phages are bacterial viruses that infect bacteria, disrupt the metabolism and cause the lysis of the bacterial host. Phages were first discovered and reported by d’Herelle [12]. Although d’Herelle quickly proposed the concept of using phages as an antimicrobial agent soon after the discovery of phages, the first-ever publication on usage of phage in treating bacterial infection was reported by Bruynoghe and Maisin [13]. D’Herelle only reported his own experimental study in 1926 [14]. Phage as a therapeutic agent fulfills almost all the criteria listed for a good antimicrobial agent [15]. The characteristics that make phages an antimicrobial agent of choice as compared to antibiotics include that phages: i) are highly specific, ii) do not cause microbial imbalance, iii) are able to replicate at the site of infection, iv) are able to reach areas with poor blood circulation, and v) do not cause serious side effects [16–18]. In conclusion, it is obvious that phages have certain advantages over antibiotics.

Currently, some phage therapeutic products have been licensed and approved for human application in some Eastern Europe countries. One of the phage preparations approved for human application in Georgia is the Phage BioDerm, a biodegradable, non-toxic polymer impregnated with bacteriophages together with antibiotics (ciprofloxacin and benzocaine) [19]. The bacteriophages contained in the polymers include lytic phages against *Pseudomonas aeruginosa*, *Escherichia coli*, *Staphylococcus aureus*, *Streptococcus* and *Proteus*. In a case study, Phage BioDerm was used in treatment of ulcers and wounds, with a successful rate of 70% on patients who failed to respond to conventional therapy [19]. Experimental phage therapy against other antibiotic-resistant bacteria such as *P*. *aeruginosa*, *Yersinia pestis* and *Burkholderia* *cepacia* has also shown promising results in mouse models [20–23]. These results suggest that phages are a viable potential alternative for antibiotics.

Studies on phages of *B*. *pseudomallei* have reported several phages with broad infectivity on *Burkholderia* species other than *B*. *pseudomallei* [24–27]. However, to date, no study on phage therapy for *B*. *pseudomallei* has been reported. In order to investigate the potential of the phage therapy for melioidosis, this study was performed to determine the effects of a novel phage C34 isolated from seawater on *B*. *pseudomallei*-infected cells.

## Materials and Methods

### Bacterial Strains

A total of 43 *B*. *pseudomallei* strains used in this study were obtained from the Medical Microbiology Diagnostic Laboratory, University of Malaya Medical Centre (UMMC) Kuala Lumpur and Hospital Tengku Ampuan Afzan (HTAA) Kuantan, Pahang. These strains were collected over the years from 1997–2013 and identified as *B*. *pseudomallei* using API 20NE (Biomerieux, France) and PCR using in-house primers [28]. In addition, *P. aeruginosa* ATCC 9027, two clinical isolates of *B. cepacia* (CQK and CYH), and *Burkholderia thailandensis* E264 were also screened as potential hosts. All the bacterial strains were cultured overnight using Luria-Bertani (LB), agar or broth, at 37°C.

### Phage Isolation

Phage C34 was isolated from the seawater sample collected from Port Dickson, (Negeri Sembilan, Malaysia) using *B*. *pseudomallei* clinical isolate strain CMS as the host. No permission was required to collect the sample from the site and the study did not involve endangered or protected species. A previously described phage enrichment method was used to enrich and isolate the phage from the seawater sample collected [29]. In brief, the sample collected was added into double strength LB broth that was inoculated with an overnight culture of *B*. *pseudomallei* strain CMS and incubated overnight at 37°C with shaking (180 rpm). The lysate was centrifuged and filter sterilised using a 0.45 μm syringe filter. The presence of phage was detected by spotting 10 μl of the lysate on an agar inoculated with an overnight culture of *B*. *pseudomallei* strain CMS. A single plaque was then picked and purified three times [30].

### Determination of Phage Host Range

All of the bacterial strains used in this study were tested to determine the host range of phage C34. An overnight culture of each bacterial strain was inoculated into 3 ml of molten soft LB agar (0.5%) overlaid on 1.5% solid LB agar. The phage was then spotted onto the solidified soft agar and incubated overnight at 37°C. Formation of a clear zone on the bacterial lawn was recorded as a positive result.

### Preparation of High Titre Phage Lysate

Phage C34 was inoculated into 100 ml of *B*. *pseudomallei* strain CMS culture with OD_600nm_ of 0.6. The culture was incubated at 37°C for 2–4 hours, with shaking at 180 rpm until lysis occurred. A few drops of chloroform were added to complete the lysis. The lysate was centrifuged and sterilised using a 0.22 μm filter membrane and the phages were precipitated using polyethylene glycol (PEG-6000) [31]. Briefly, the lysate was mixed with PEG-6000 (final concentration: 10%, w/v) and the phage was harvested by centrifugation at 18,500 × g for one hour. The pellets were suspended in phosphate buffered saline (PBS) and stored at 4°C. The phage titre was then determined [30].

### Transmission Electron Microscopy (TEM)

Negative staining method was used to observe the morphology of phage C34 under TEM [32]. The concentrated phage was deposited on a carbon-coated copper grid and stained with 2% phosphotungstic acid. Excessive solution was drained using filter paper and the grids were observed using Hitachi HT7700 TEM.

### One-step Growth Parameter

The physiological characteristics of phage C34 were determined using one-step growth curve experiments as described previously [33]. In brief, approximately 1 × 10^8^ colony forming unit (CFU/ml) of *B*. *pseudomallei* strain CMS were infected by phage C34 at an multiplicity of infection (MOI) of 1 (phage:bacteria). The mixture was incubated at 37°C for 10 min before centrifugation and resuspension of pellet in 10 ml of LB broth. Two hundred microliters of culture were taken at predetermined intervals, half of which was treated with 1% (v/v) chloroform to release the intracellular phage and plated on the bacterial lawn for the latent period determination. The other half was immediately diluted and plated to measure the phage titer. The experiment was repeated three times.

### Temperature Stability Test

Approximately 1 × 10^8^ PFU/ml of phage was suspended in PBS and incubated at 37°C, 65°C and 90°C. At predetermined intervals, the phage titre was determined [34].

### Bacterial-phage Challenge Test

Approximately 1 × 10^8^ CFU/ml of *B*. *pseudomallei* strain CMS was infected by phage C34 at MOI of 10, 1 and 0.1 [35]. Viable bacterial counts and plaque forming units of phage C34 were determined every hour. In addition, growth curves were generated using absorbance readings at 570 nm that was recorded hourly for 24 hours. Uninfected *B*. *pseudomallei* strain CMS was used as a negative control and *B*. *pseudomallei* strain CMS treated with 500 μg/ml of kanamycin was used as the positive control.

### Isolation and Generation of Growth Curve of Bacteriophage Insensitive Mutants (BIMs)

Viable bacterial counts of the bacterial culture used in absorbance readings was determined at the end of the 24 hours experiment. During each replicate, five independent colonies with distinct morphology from the parental strain were isolated (in total, n = 15). The isolated bacteria were tested for phage resistance by streaking the bacteria across a perpendicular line of phage on an agar plate. A bacterium was considered as BIM if no inhibition of growth was observed after overnight incubation [21, 36]. Growth curves of the BIMs were generated using absorbance readings at 570 nm that was recorded hourly for 24 hours. The parental strain, *B*. *pseudomallei* strain CMS served as the control.

### Frequency of Emergence of BIMs

*B*. *pseudomallei* strain CMS of known cell numbers was infected by phage C34 at the MOI of 10 (phage:bacteria). The mixture was inoculated into 3 ml of molten soft LB agar (0.5%) and then overlaid on 1.5% solid LB agar. The agar plate was incubated overnight at 37°C and the colonies were counted. The experiments were performed in triplicate and the frequency of BIM was determined by dividing the number of colonies formed with the original cell numbers inoculated [37].

### A549 Cells Survivability Assay

Human lung epithelial cells, A549 were obtained from American Type Culture Collection (Rockville, Maryland) and cultured in complete RPMI medium 1640 (Gibco® RPMI 1640 medium (GIBCO, United Kingdom) supplemented with 10% fetal calf serum). The cells were grown at 37°C and 5% CO_2_ to form a confluent monolayer.

Approximately 2 × 10^4^ A549 cells were infected with 2× 10^5^ CFU of *B*. *pseudomallei* strain CMS grown to mid-log phase. Concurrently, for the purpose of investigating the prophylactic effect of phage C34, 2 × 10^4^ A549 cells were pre-treated using 2 × 10^7^ phage C34 particles overnight and washed three times with PBS before proceeding with the infection with 2 × 10^5^ CFU of *B*. *pseudomallei* strain CMS grown to mid-log phase. The infection of A549 cells was performed for two hours at 37°C. The infected cells were washed three times with PBS, and then treated with phage 2 × 10^7^ phage C34 particles in RPMI complete medium supplemented with 500 μg/ml of kanamycin (to eliminate any remaining extracellular bacteria). The assay plates were incubated in the presence of 5% CO_2_ at 37°C overnight. The cells were then washed three times with PBS to eliminate the dead cells and the survivability was determined using modified crystal violet cell viability assays [38]. Briefly, crystal violet solution (0.1%) was added to assay plates for three minutes, removed, and the cells were washed three times with distilled water and air-dried. Absolute ethanol was added to resolubilise the stain and the absorbance was measured at 570 nm. Untreated but infected A549 cells were used as positive control while uninfected A549 cells were used as negative control. Uninfected but treated A549 cells were used to determine the toxicity of phage C34. In order to eliminate the possibility of non-specific immune response induced by the impurities in phage preparation, heat-killed phage (65°C, 40 minutes) was used to treat the A549 cells prior to infection.

Viability of A549 cells was calculated using the formula below:

$$\frac{B}{A}\times 100\%$$

Where

A = Absorbance of negative control,

B = Absorbance of sample

### Experimental Phage Therapy in Mice

Experiments were performed on specific-pathogen-free BALB/C mice (aged six to eight weeks, female). The animals were maintained under specific-pathogen-free conditions, housed in sterile cages with a bedding of paper shavings, subjected to a 12-hour light/dark cycle, and fed a diet of commercial pellets, with water provided *ad libitum*. The animal work was performed with approval from University of Malaya Institutional Animal Care and Use Committee (File no: PAT/05/11/2007/0912/WKT).

In order to explore the therapeutic effect of phage C34 in the infected animals, the mice were infected intranasally (i.n.) with *B*. *pseudomallei* strain CMS (100 CFU) using the method previously described by Conejero *et al*. [39] with slight modifications. Three groups, each consisting of 15 mice were used. The first group was intraperitoneally (i.p.) administered with 2 × 10^8^ PFU of phage C34 in 100 μl of PBS at 24 hours prior to infection. Likewise, 2 × 10^8^ PFU of phage C34 was administered via i.p. route into the second group, two hours post-infection. The third group served as the control (infected but not administered with phage C34). The mice were then monitored daily for disease symptoms, to a maximum of 14 days. The unhealthy mice were euthanized according to humane end points (defined by signs of moribundity: hunched posture, lack of responsiveness to manual stimulation and inability to eat/drink). Euthanasia of mice was performed via isoflurane exposure. Spleens were harvested from the euthanized surviving mice and examined for signs of infection such as macroscopic lesions and splenomegaly.

A second set of animals was used to examine the antimicrobial effect of phage C34 on *B*. *pseudomallei* infected mice. The experimental groups and procedure were the same as described above. The number of animals was 18 mice per group. The bacterial burden at mice tissues (n = 6 per group per day) was enumerated by harvesting the organs from the mice at day 1, 2 and 3 post-infection. Briefly, blood was collected from the mice via cardiac puncture using syringe rinsed with EDTA. Lung, liver and spleen of the mice were obtained aseptically and homogenised in sterile PBS using a tissue homogeniser. Blood and homogenised tissue samples were plated onto Ashdown’s agar and incubated at 37°C for 48 hours. Presence of BIM was tested by streaking ten colonies from each group across a perpendicular line of phage on an agar plate.

The presence of phage C34 in the mice administered i.p. with 2 × 10^8^ PFU of phage C34 in 100 μl of PBS compared to control was examined in a third set of animals. There were three groups of animals; phage C34 administered 24 hours prior to infection with *B*. *pseudomallei* strain CMS, phage C34 administered two hours post-infection and phage C34 administration into mock-infected mice (control). The mice were then euthanized at day 1, 2 and 3 post-infection (n = 6 per group per day) and the phage titre in the blood, lung, liver and spleen of the mice was determined.

## Results

### Isolation and characterisation of phage C34

**Fig 1:**
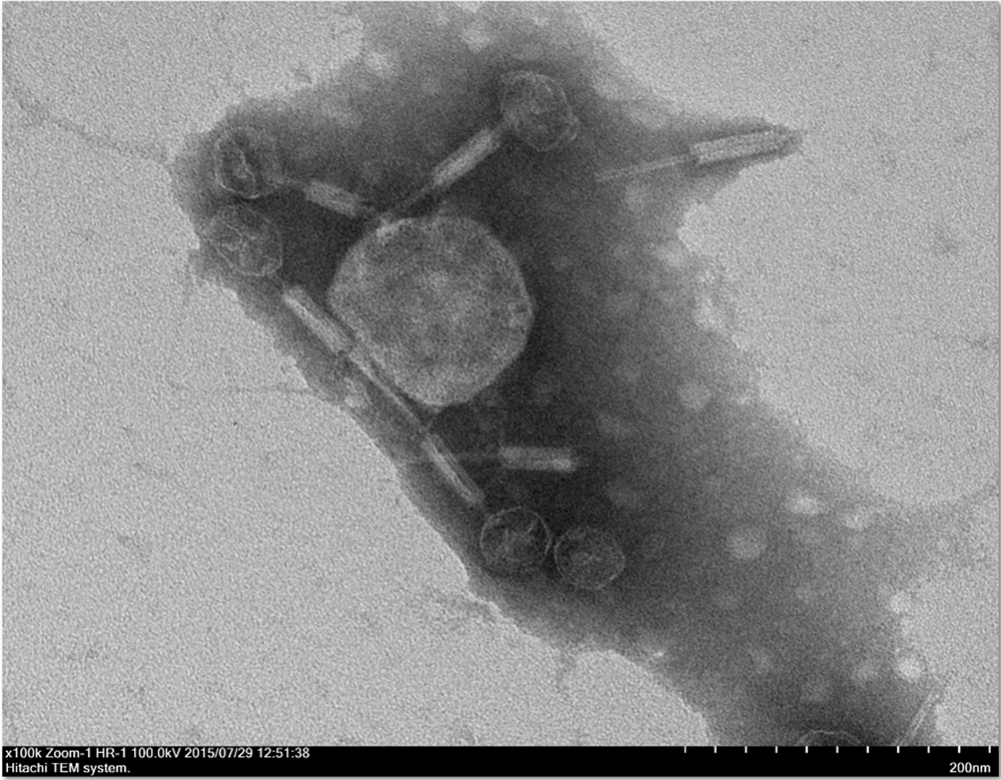
Transmission electron microscopy image of phage C34. The phages show an icosahedral head with contractile tail, which is a typical morphology of the family Myoviridae. Magnification: x100000.

**Fig 2:**
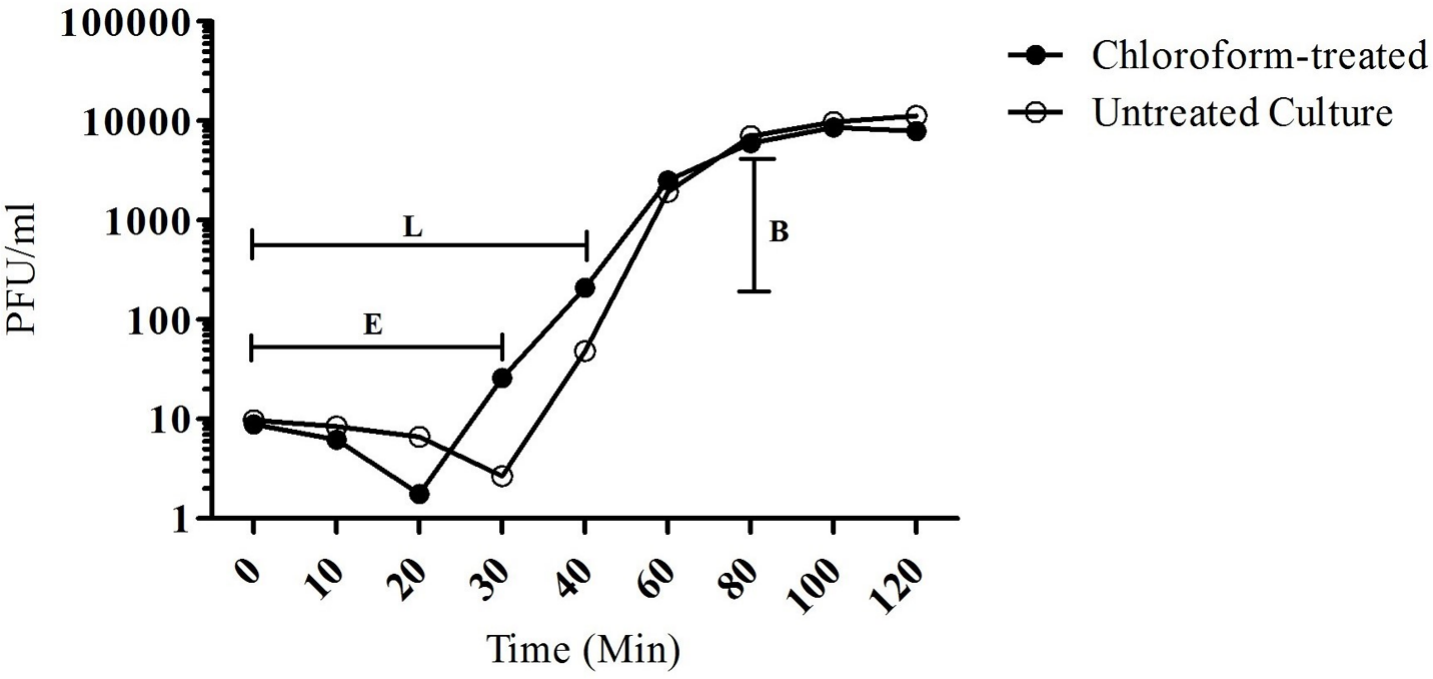
One step growth curve of phage C34. The infection cycle of phage C34 propagating on *B*. *pseudomallei* strain CMS. E, eclipse period (30 minutes); L, latent period(40 minutes); B, burst size of the phage (234).

Phage C34 was isolated from a seawater sample collected from Port Dickson, Negeri Seremban. Morphogically, the newly isolated phage C34 has a head measuring 50 nm in diameter and a contractile tail measuring 138 nm in length (Fig 1). The observed head and contracted tail places phage C34 into the family Myoviridae. Phage C34 was found to be able to form clear zone on 53.5% of the *B*. *pseudomallei* clinical isolates (23/43) but not the *P*. *aeruginosa* ATCC 9027, *B*. *cepacia*, and *B*. *thailendensis* tested. The eclipse and latent period of phage C34 were 30 and 40 minutes, respectively while the burst size of phage C34 was calculated to be 234 (Fig 2). It was found that phage C34 was relatively stable at 37°C as phage titre remained the same after 30 minutes as compared to higher temperatures. At 65°C, the phage titre demonstrated a near linear regression (R^2^ = 0.9430) and the rate of regression was -0.142 log PFU/ml/min. However, at 90°C, the phage titre became too low to be detected after 5 minutes, indicating that phage C34 is heat labile. On the contrary, phage C34 was noted to be stable at 4°C with a reduction of less than 1 log PFU/ml within a period of 2 months.

### Lytic activity of phage C34 *in vitro*

**Fig 3:**
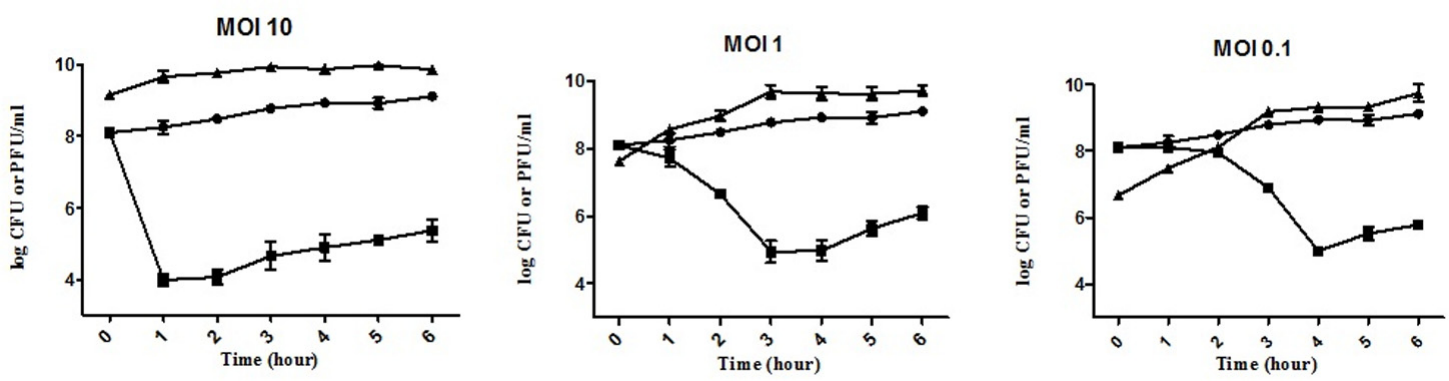
Bacterial count of *B*. *pseudomallei* over the course of 6 hours. Control (●), *B*. *pseudomallei* infected by different MOIs (■) and the corresponding phage titre of C34 (▲).The graph shows averages for three independent assays.

*B*. *pseudomallei* strain CMS were challenged by phage C34 with the MOI of 10, 1 and 0.1 (phage:bacteria). The result showed that MOI of 10 was the most potent MOI, with killing of bacteria occurred as early as the first hour after the addition of phage and a reduction of 4 log CFU as compared to the initial inoculum (Fig 3). At lower MOIs (1 and 0.1), the number of bacteria was reduced to 4.95 ± 0.33 log CFU/ml at 3 hours post-challenge and 5 ± 0.08 log CFU/ml at 4 hours post-challenge, respectively. In all cases, the number of bacterial count gradually increased after the killing/reduction point. The growth curve generated using optical density was found to complement the time kill curve. The application of phage C34 resulted in the reduction of bacterial growth, which was represented by the drop in the OD reading (result not shown). Similarly, the higher the MOI, the earlier the killing of the bacteria occurred. Growth of the phage-infected *B*. *pseudomallei* strain CMS was found to be increasing again following the drop, albeit at a slower rate as compared to the uninfected control.

### Isolation of BIMs and its frequency of emergence *in vitro*

**Fig 4:**
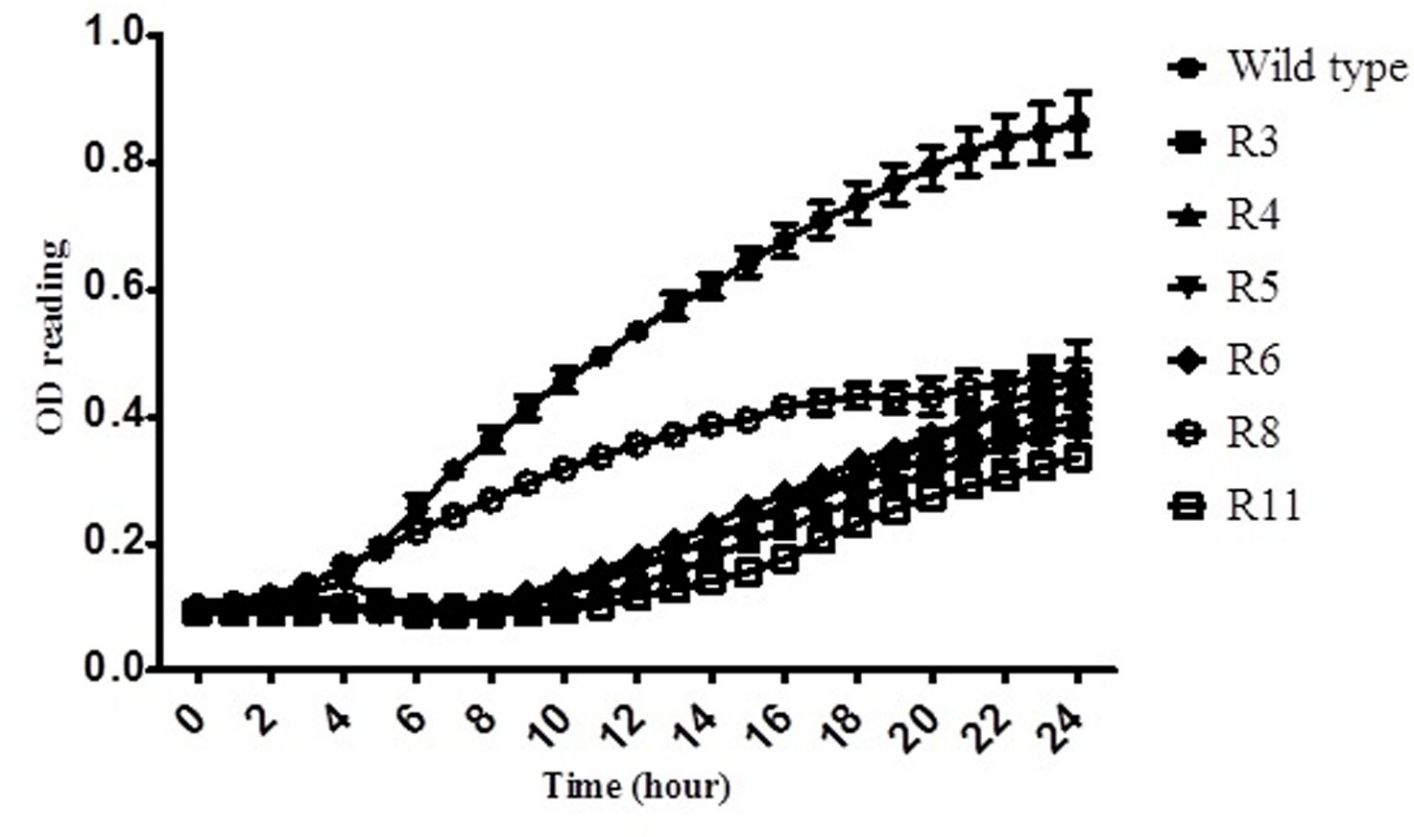
Growth curve of *B*. *pseudomallei* strain CMS (wild type) and the phage-resistant isolates. Six of the isolates appeared to grow slower than the parental strain. The graph shows averages for three independent assays. Error bars indicate the standard error of the averages.

At the end of the time-kill experiment, a total of 15 colonies with distinct morphology (mucoid) from the wild type strain were isolated and tested for phage resistance. Seven of the isolates were found to be resistant to infection by phage C34 (result not shown). The growth curves of these isolates were generated using absorbance reading at 570nm. It was shown that six out of seven isolates grew slower than their parental strain, *B*. *pseudomallei* strain CMS (Fig 4). Frequency of the emergence of BIM for phage C34 was found to be 2.3 × 10^−5^ ± 7.2 × 10^−6^.

### Experimental phage therapy *in vitro*

**Fig 5:**
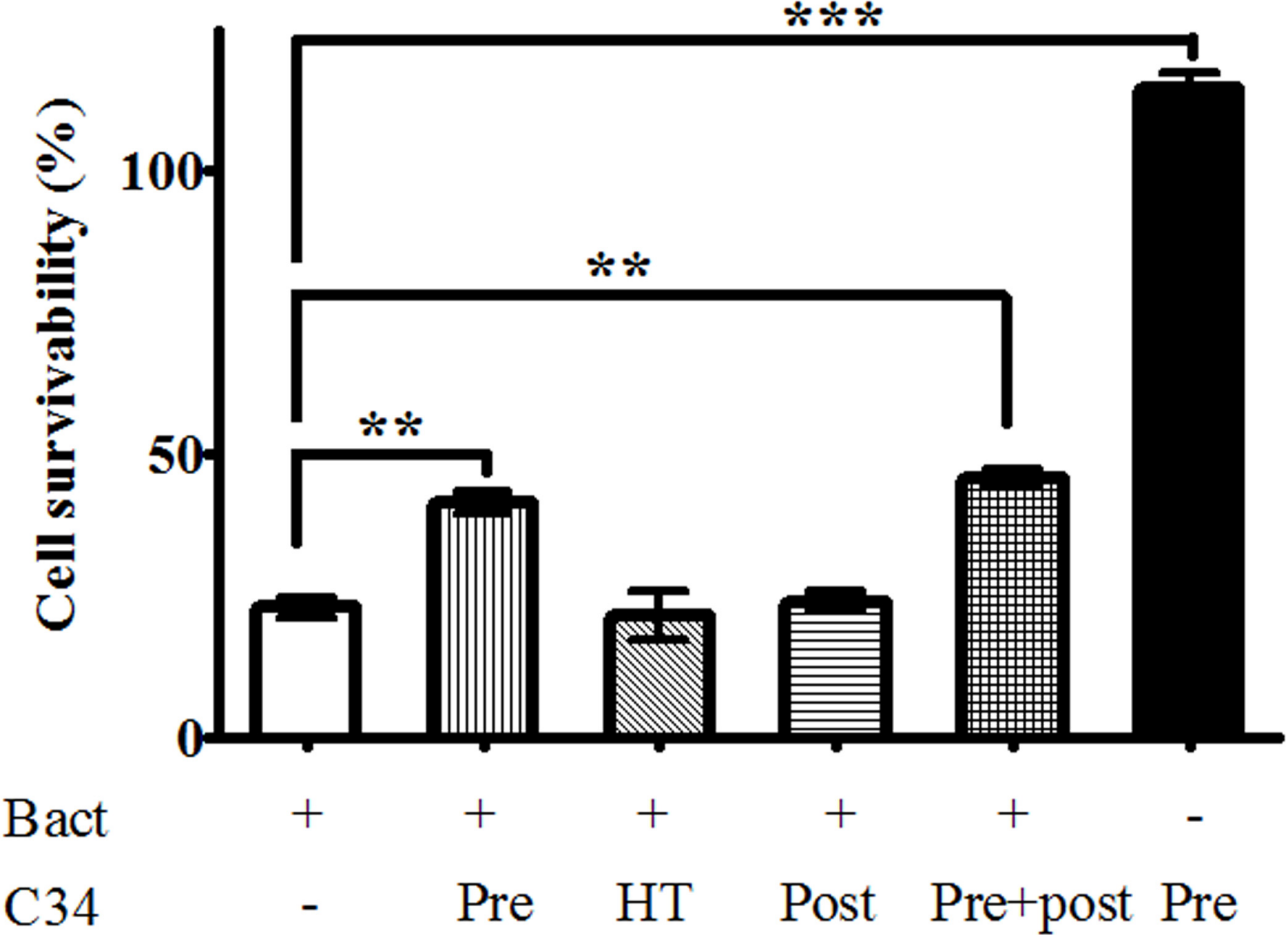
Survivability of infected A549 cells. The survivability of infected A549 cells which received pre-infection and pre + post-infection treatment was significantly higher than the control. Survivability of non-infected A549 cells was calculated as 100% and thus not shown in the figure. The results are the averages of three independent assays. Pre: Pre-infection treatment; HT: Pre-infection treatment using heat-killed phage; Post: Post-infection treatment; Pre+post: Pre-infection and Post-infection treatment.

In order to evaluate the protective effect of phage C34 against *B*. *pseudomallei* infection, *B*. *pseudomallei* strain CMS was used to infect human epithelial lung cells, A549 and the infected A549 cells were treated using 2 × 10^7^ PFU of phage C34. An average of 22.8 ± 6.0% of A549 cells infected with *B*. *pseudomallei* strain CMS survived after overnight infection (Fig 5). In this study, infected A549 cells which received post-infection treatment with 2 × 10^7^ PFU of phage C34 did not show any increase in the cell survivability. However, it was found that the survivability of infected A549 cells increased from 22.8 ± 6.0% (untreated control) to 41.6 ± 6.8% (pre-infection treated) when the A549 cells were pre-treated with 2 × 10^7^ PFU of phage C34 prior to infection by *B*. *pseudomallei* strain CMS. The A549 cells which received both pre- and post-infection treatment did not show any significant differences in the survivability of infected A549 cells when compared with the pre-infection treated control. No significant differences were found between the survivability of the negative control (uninfected A549 cells) and the uninfected cells which received phage treatment. The result suggested the application of phage C34 (up to 2 × 10^7^ PFU) did not affect the survivability of A549 cells. Pre-infection treatment using heat-killed phage preparation also did not increase the survivability of A549 cells.

### Therapeutic efficacy of phage C34 against mouse intranasal infection

**Fig 6:**
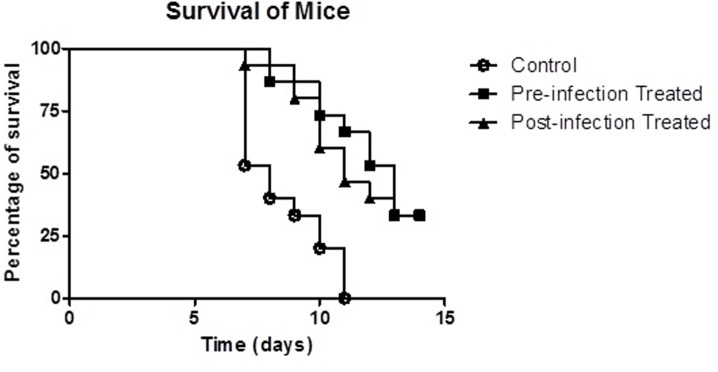
Mortality of *B*. *pseudomallei* strain CMS-infected mice. Mice which received treatment 24 hours before the infection (■) or 2 hours post-infection (▲). Control mice without any treatment (○) were 100% moribund at day 11 while 33% of the mice (n = 5) in both of the treated groups survived at the end of 14 days.

To elucidate the effect of phage C34 on *B*. *pseudomallei*-infected mice, we monitored the survivability of 15 female BALB/c mice infected intranasally with 100 CFU of B. pseudomallei strain CMS. All control mice displayed symptoms of disease such as onset of fever and lethargy upon three days of infection while the treated mice only started to show symptoms at day five. The application of 2 × 10^8^ PFU phage C34 via i.p, 24 hours prior to infection and 2 hours post-infection significantly protected the infected mice compared to the controls (p<0.001). Both of the treatment groups demonstrated similar percentage of survival (33.3%) with 5 mice surviving at the end of the experiment, respectively (Fig 6). Log rank (Mantel-Cox test) statistical analysis also revealed that there was no significant difference between the survival curves of pre- and post-infection treated groups (p = 0.7006). All of the control mice were moribund at day 11. The median of survival was 8 days for control mice, 13 days for pre-infection treated mice and 11 days for post-infection treated mice. In the process of organ harvesting, it was found that macroscopic lesions were present on the spleen of the mice and signs of splenomegaly (spleen enlargement). The results suggested that treatment using phage C34, regardless to the time of application, was able to provide partial protection towards *B*. *pseudomallei* infection.

**Fig 7:**
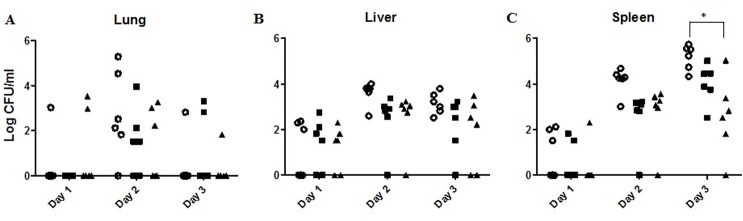
Bacterial burdens in lung, spleen, and liver of mice. Control (○), mice treated with i.p. phage treatment (2 × 10^8^ PFU of phage C34), administered 24 hours before the infection (■) or 2 hours post-infection (▲). The numbers of viable bacteria (CFU) were enumerated from the organs of mice on day 1, 2 and 3 post-infection (A-C). On day 3, the bacterial burden of mice which received post-infection treatment was significantly lower than that of the control in spleen tissues only.

No viable bacterium was detected from the blood of the mice throughout the first three days post-infection period (data not shown). Bacteria could only be detected in the lungs of less than half (50%) of the infected mice one day and three days post-infection, whereas during day two post-infection, it was found that the average bacterial load in the lungs of the control mice was higher than that of the pre- and post-infection treated group (log 2.71 ± 1.92 CFU/ml vs log 1.51 ± 1.47 and log 1.42 ± 1.59 CFU/ml) (Fig 7). However, the differences between these averages were not statistically significant (p>0.05). In the liver, all three groups showed similar average bacterial load on day one, with log 1.11 ± 1.22, 1.37 ± 1.14 and 1.19 ± 0.97 CFU/ml for the control, pre- and post-infection treated group, respectively. The average bacterial load of control group was higher than the bacterial load of both treated group for days two and three. However, the differences between the averages were again not statistically significant (p>0.05). In the spleen, all three groups showed similar average bacterial load on day one. The average bacterial load of control group was higher than that of both treated group for days two and three, with at least one log of inhibition observed in the treated groups as compared to the control group. Statistical analysis revealed that the differences between the groups at day two were not significant. However, on day three, the average bacterial load in the spleen of the post-infection treated mice is significantly lower than that of the control mice group (p<0.001), whereas it is not significant for the pre-infection treated group (p>0.05).

**Fig 8:**
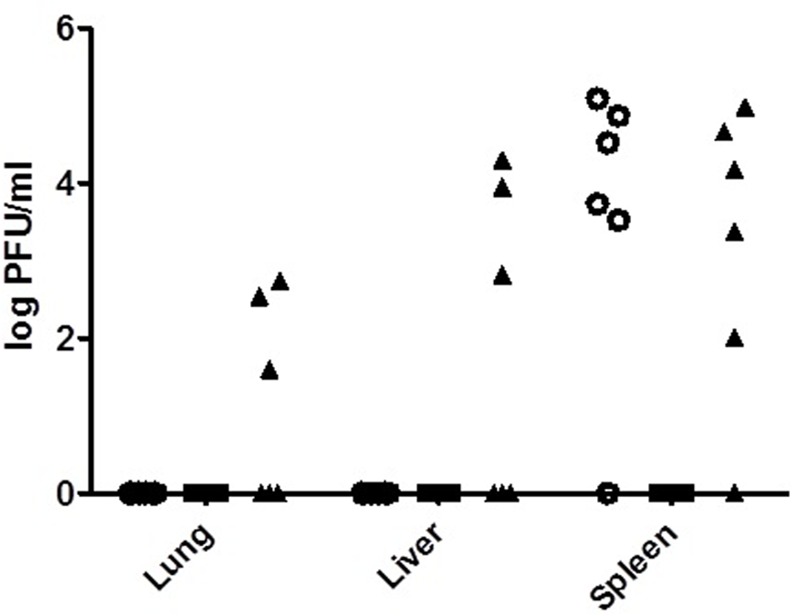
Recovery of phages from the mice tissues following i.p. administration of 2 × 10^8^ PFU phage C34. Presence of phages were examined at 24 hours post-injection in the mock-infected mice (○), at 24 hours after the infection in pre-infection treated mice (■) and 24 after the infection in post- infection treated mice (▲). Phages were recovered from the lung, liver and spleen of the mice which received post-infection treatment, and from the spleen of the mock-infected mice.

The presence of phage in the mice organs was determined by administering 2 × 10^8^ PFU of phage C34 via i.p., 24 hours prior to infection and 2 hours post-infection into 6 BALB/C mice. Control mice were mock-infected with PBS. Blood, lung, liver and spleen of the mice were harvested daily for 3 days and titre for the presence of phage. No phage was detected in the blood of mice in all of the groups throughout the three days of experiment (data not shown). For control mice, an average of log 4.35 ± 0.69 PFU/ml of phage C34 was recovered from the spleen of the mice (n = 5) while none was detected from the lungs and liver on day one post-treatment (Fig 8). No phage was detected from all tissues for day two and three post-treatment (data not shown). As for the pre-infection treated group, no phage was recovered from all tissues for all three days (data not shown). In the post-infection treated group, phage was present in the lungs, liver and spleen of the infected mice one day post-infection. However, only half of the mice injected (n = 3) were found to be bearing the phage C34 in the lungs and liver, with an average of log 2.29 ± 0.61 and 3.69 ± 0.77 PFU/ml, respectively. In the spleen, phage C34 was detected in five of the mice with an average of log 3.83 ±1.19 PFU/ml. Statistical analysis revealed that there was no difference in the average number of phage found from the spleen between the control and post-infection treated group (p > 0.05). Similar to the control group, no phage was recovered at day two and three post-injection in both, pre- and post-infection treated groups. No mucoid colony was observed from all groups and no BIM was detected among the tested colonies.

## Discussion

To date, there has been no report of effective phage therapy for *B*. *pseudomallei*, an intracellular pathogen. However, the experiences of phage therapy on other intracellular pathogens suggest that the idea may be worthwhile. Phage therapy had been effective against some intracellular pathogens such as *Mycobacterium tuberculosiss* [40, 41], *S. aureus* [23] and *Y. pestis* [20] in experimental studies performed *in vitro* (macrophage model) and *in vivo* (mice model) using phage-infected bacteria as a vehicle to deliver the phages.

Determination of the host range is important in order to select a suitable candidate for phage therapy. Initial characterization of the host range of phage C34 was determined using spot test. It was found that phage C34 was able to form clear zones on 53.5% of the *B*. *pseudomallei* clinical isolates. This result was comparable with the report by Yordpratum *et al*. [27], whereby the phages isolated were found to have the ability to lyse 41%–78% of *B*. *pseudomallei* strains in their collection. In contrast, Gatedee *et al*. [24] reported that the phage isolated was able to lyse all tested *B*. *pseudomallei* strains. This result needs to be treated with caution since Gatedee and colleagues have only tested their phage on 11 strains of *B*. *pseudomallei*. Both the present and the study by Yordpratum *et al*. tested the phages against 43 and 63 *B*. *pseudomallei* strains, respectively. It is also interesting to note that two of the phages isolated by Yordpratum *et al*. were able to lyse non-*B*. *pseudomallei* bacteria while phage C34 was found to be strictly host specific.

In this study, we report the experimental phage therapy for *B*. *pseudomallei* in a non-phagocytic cell culture model (A549 human lung epithelial cell) in the absence of a direct phage-delivery vehicle. C34, a phage isolated from seawater sample, showed promising results, with an increase of 20% survival rate in pre-infection treated A549 cells compared with non-pre-infection treated cells in the cell survivability assay. The survivability of infected cells did not increase in the group which received both pre- and post-infection treatment. This led us to postulate that infection caused by *B*. *pseudomallei* may reduce permeabilisation or internalisation of phage C34 into A549 cells, thus limiting the effect of post-infection application. This is supported by a microarray assay conducted by our group, in which certain pathways involving endocytosis and phagocytosis were down-regulated in *B*. *pseudomallei* strain CMS-infected A549 cells (unpublished data). The efficiency of phage uptake can be investigated in future studies using immunofluorescence technique.

Nevertheless, this study indicates that the antibacterial property of the administered phage contributed to the survival of the infected mice, which is evident through the protective effect shown by phage C34 in the *in vitro* model. Furthermore, the use of heat-killed phage in pre-infection treatment of A549 cells did not increase the survivability of A549 cells. Thus, in combination with both the observation from the *in vitro* and *in vivo* model, it can be suggested that the activity of phage C34 did contribute to the therapeutic effect against *B*. *pseudomallei* infection. This hypothesis was further supported by the fact that administration of bacteriophage also reduced the bacterial load in the organs of the infected mice. The reduction in the bacterial load in spleen was particularly obvious, in which the average bacterial load in the spleen of the post-treated mice at day three was approximately two fold lower than that of the untreated controls. The observation of lesions and splenomegaly in the spleen of the surviving mice at the end of the 14-days observation period was an indication that sterile immunity was not achieved. To date, it is still challenging to achieve sterile immunity against *B*. *pseudomallei* [39]. In this study, even though 33% of the infected mice were rescued with the single injection of phage, *B*. *pseudomallei* still persisted in the organs of the surviving mice. Thus, similar to antibiotic treatment on melioidosis patients, we believe that an eradication therapy is required for total clearance of the bacteria.

Bacteriophage insensitive mutants forming mucoid colonies emerged upon post-exposure to phage C34 *in vitro*. In the absence of phage, six of these isolates grew slower as compared to the wild type. A review of literature shows that mucoid or alginate production of bacteria contributed to phage resistance [42–44] and lowered the growth rate of the BIMs [21]. Thus, the presence of mucoid colony from the organs of the phage-treated mice was much anticipated. However to our surprise, neither mucoid colony nor BIM was observed amongst the bacteria isolated from the organs of the phage-treated mice. We believe that any BIMs which emerged upon phage exposure *in vivo* was eliminated by the mice immune system as mucoid production was often linked with fitness cost which potentially reduces the virulence of the bacteria [45, 46]. Future studies on the BIMs will includes phage receptor on both wild type and the BIMs, phage-host interaction, stability of BIMs and a full-fletched study on the virulence of BIMs.

Phages administered to experimental animal models have been known to be sequestered in the spleen and cleared from the circulation in a short period [45, 47, 48]. In this study, administration of phage C34 into control mice via the i.p. route also showed similar results where phage can only be detected in the spleen of the control mice, whilst phage can be recovered from the spleen as well as the lung and liver of half (50%) of the infected mice, one day post-infection. In both groups of control and infected mice, phages were only detected at day one post-injection and post-infection, respectively. This result is in agreement with previous studies which reported phage titre in the lungs of the infected mice to be significantly higher than the control mice, and this was attributed to the *in vivo* replication of the phages [49, 50]. Likewise, in this study, the phages could be circulated and retained (temporarily) in the infected organs in the presence of host bacteria. No phages were recovered in the pre-treated group. This is likely because the presence of phages were examined on days one to three post-infection, where day one post-infection was already day two post-phage injection. This result complemented the results of the control and post-treated group where no phages were recovered at day two post-injection. In the absence of host bacteria, the phages would be sequestered in the spleen and cleared from the mice system thereof. The use of selection method to select for mutant phages with prolonged circulation in mice system [51] may be able to improve the persistence of phage C34 in the circulatory system, and thus improving its efficacy of the phage therapy.

In conclusion, we isolated phage C34 from seawater and successfully tested its efficacy on *B*. *pseudomallei*-infected A549 epithelial cells and BALB/c mice. In this study, we report the potential of phage C34 to provide protection against *B*. *pseudomallei* challenge. However, further studies are required to examine the pharmacokinetics of this phage therapy system and a few methods were suggested to improve the efficacy of the treatment.
